# Overview of Wnt/β-Catenin Pathway and DNA Damage/Repair in Cancer

**DOI:** 10.3390/biology14020185

**Published:** 2025-02-11

**Authors:** Silvina B. Nadin, F. Darío Cuello-Carrión, Niubys Cayado-Gutiérrez, Mariel A. Fanelli

**Affiliations:** 1 Laboratorio de Biología Tumoral, Instituto de Medicina y Biología Experimental de Cuyo (IMBECU), Universidad Nacional de Cuyo, Consejo Nacional de Investigaciones Científicas y Técnicas (CONICET), Centro Científico Tecnológico (CCT), Mendoza 5500, Argentina; 2 Laboratorio de Oncología, IMBECU, CONICET, CCT, Mendoza 5500, Argentina; dcuello@mendoza-conicet.gob.ar (F.D.C.-C.); ncayado@mendoza-conicet.gob.ar (N.C.-G.); mfanelli@mendoza-conicet.gob.ar (M.A.F.); 3 Cátedra de Bioquímica e Inmunidad, Facultad de Ciencias Médicas, Universidad Nacional de Cuyo, Mendoza 5500, Argentina

**Keywords:** Wnt/β-catenin, DNA repair, cancer

## Abstract

The Wnt/β-catenin pathway takes part in important cellular processes in tumor cells, such as gene expression, adhesion, and survival. The canonical pathway is activated in several tumors, and β-catenin is its major effector. The union of Wnt to the co-receptor complex causes the inhibition of GSK3β activity, thus preventing the phosphorylation and degradation of β-catenin, which accumulates in the cytoplasm, to subsequently be transported to the nucleus to associate with transcription factors. The relationship between Wnt/β-catenin and DNA damage/repair mechanisms has been a focus for the last few years. Studying the Wnt/β-catenin network interactions with DNA damage/repair proteins has become a successful research field. This review provides an overview of the participation of Wnt/β-catenin in DNA damage/repair mechanisms and their future implications as targets for cancer therapy.

## Simple Summary

Cancer is the main cause of death globally. The dysregulation of the Wnt/β-catenin pathway is widely recognized for its role in tumorigenesis, but it is also associated with critical homeostatic functions within cells. In recent years, significant advancements have been made in the progress of targeted therapies for various types of cancer. Consequently, it is essential to dig deep into our knowledge of how the Wnt/β-catenin pathway interacts with other important mechanisms, such as DNA repair. Precision medicine aims to provide cancer patients with personalized and combined therapies that exhibit reduced cytotoxicity and help overcome resistance. This overview will examine the relationship between the Wnt/β-catenin pathway and DNA repair mechanisms.

## 1. Introduction

Cancer constitutes one of the main causes of mortality in the world. Genomic stability maintenance is crucial to prevent this disease and more accurately it was considered a cancer hallmark [1]. The DNA molecule sustains human life and cellular processes; however, it is constantly threatened by environmental and endogenous agents that cause various forms of DNA damage including base alterations, DNA double helix distortion, and strand breaks, among others [2]. Fortunately, the capacity of the cells to repair DNA damage, and a correct DNA repair function, maintains the cellular homeostasis preventing carcinogenesis, genetic disorders, and a diversity of diseases [3]. The DNA repair pathways include an intricate network of mechanisms for repairing specific DNA lesions. They are divided into five principal mechanisms beginning with the DNA damage response (DDR), base excision repair (BER), nucleotide excision repair (NER), mismatch repair (MMR), and double-strand break repair (that includes homologous recombination and non-homologous-end joining). These pathways are usually complex, and coordinated, have multiple steps, and consist of several proteins with catalytic activities [4].

Targeted therapies, or precision medicine, can use DNA repair defects that are characteristics of cancer cells [5]. Beyond repairing gene mutations, the genomic imprint of somatic mutations in cancer can be applied to diagnose deficiencies in DNA repair deficiencies and select specific molecules for therapy [6,7].

In addition to DNA repair mechanisms, the Wnt signaling pathway is an intricate network of proteins implicated in cancer hallmarks, which participates in embryonic development, tissue development, tumorigenesis, and cancer progression. Moreover, this pathway participates in many cellular processes such as differentiation, cell migration, apoptosis, antineoplastic drug resistance, and genomic stability. The canonical Wnt/β-catenin signaling pathway exerts a crucial role in other cellular functions including radioresistance in cancer cells [8,9].

This overview provides insights into the relationships between DNA repair and the Wnt/β-catenin pathway, and their implications in cancer progression and treatment. Accordingly, understanding the interplay between Wnt/β-catenin and DNA repair mechanisms is important for developing precision-targeted cancer therapies, a valuable scenario to enhance prognosis in clinical oncology.

## 2. DNA Damage Response and DNA Repair Mechanisms

DNA is constantly exposed to damage from external and/or internal agents and if lesions are unrepaired or incorrectly repaired the alterations or mutations affect cell survival or genome integrity [10]. Fortunately, cells are composed of an efficient network of molecules including sensors of DNA damage, mediators of the response, transducers, and downstream effectors, known as DNA damage response (DDR). DDR involves a signaling cascade from DNA damage sites which ultimately activates the cell-cycle checkpoints to allow for repair or triggers apoptosis or senescence. The DDR is primarily executed by the phosphoinositide 3 kinase proteins (PI3Ks): ataxia-telangiectasia-mutated (ATM), ATM- and RAD3-related (ATR), and DNA-dependent protein kinase (DNA-PK) and by the poly (ADP-ribose) polymerase (PARP) proteins. DNA-PK and ATM detect and repair DNA double-strand breaks (DSBs) Ref. [11], and PARP1 and ATR are activated in response to DNA single-strand breaks (SSBs) [11,12]. The downstream effectors are CHK1 and CHK2 (checkpoint kinases 1 and 2) and histone H2AX at DNA damage sites. H2AX is phosphorylated to originate γH2AX, one of the first events in DDR. γH2AX amplifies the DDR signaling and recruits proteins [13]. CHK1 and CHK2 phosphorylate P53, promoting DNA repair or apoptosis [14]. In brief, CHK kinases, such as CDKs (cyclin-dependent kinase), CDC25, and P53, activate the effectors for cell-cycle control and DNA repair. The tumor suppressor TP53 is the most commonly mutated gene in human cancers, playing critical roles in cell proliferation and genome stability, thus coordinating the cell-cycle arrest and/or DNA repair machinery [15]. Cellular fate depends on the severity of DNA damage, so cells with excessive or unrepairable DNA undergo cell death [16]. Several studies have demonstrated that DDR is associated with carcinogenesis and cancer progression, including radiotherapy and chemotherapy efficiency [17].

### 2.1. DNA Repair Mechanisms

Cells are continuously exposed to diverse types of DNA damage which can be repaired by specialized mechanisms including direct repair (DR), base excision repair (BER), nucleotide excision repair (NER), mismatch repair (MMR), homologous recombination (HRR) and non-homologous-end joining (NHEJ). The functionality of these repair pathways is essential to maintain genome stability and prevent carcinogenesis [18]. A brief description of each DNA repair mechanism will be provided below.

#### 2.1.1. Direct Repair

The direct repair mechanism in humans specializes in correcting the DNA damage induced by alkylating agents. DNA alkylation damage can occur from exposure to external agents such as N-methyl-N’-nitro-nitroso-guanidine (MNNG) or endogenous sources. The most frequent lesion of this type is O6-methylguanine (O6-meG) which is recognized by O6-meG-DNA-methyltransferase (MGMT) [19]. The expression of MGMT is characteristically high in tumors and years ago emerged as a target for cancer therapy [20]. However, MGMT deficiencies can occur because of promoter methylation [21].

#### 2.1.2. Base Excision Repair

The base excision repair (BER) corrects base damage: oxidative DNA damage, alkylation, deamination, and abasic sites in the double helix. The repair of these lesions begins with the recognition step of specific enzymes known as DNA glycosylases. BER has two sub-pathways: short patch-BER and long patch-BER [22]. The DNA glycosylase excises the DNA damage, hydrolyzing the N-glycosidic bond between the nitrogenous base and deoxyribose, leaving an apurinic/apyrimidinic (AP) site. Approximately, eleven glycosylases are known, which recognize specific DNA base damage with overlapping characteristics [23]. Then, an AP endonuclease incises the AP site creating a nick, allowing DNA polymerase β to change the damaged base (and a lyase), in the short patch-BER. Instead, if the lesion site consists of 2–11 nucleotidesm other components, including PCNA and FEN1 endonuclease, intervene in the long patch-BER [23]. BER proteins can be altered in several human cancers (germline polymorphisms, germline, and somatic mutations). Furthermore, the expression status of the BER genes is a promising prognostic and predictive biomarker in cancer [22]. Different BER inhibitors for DNA glycosylases, APE1, PARP1, End Processing Enzymes, Gap Filling Enzymes, and Nick Sealing Enzymes have been developed, and clinical trials are evaluating their effectiveness [22].

#### 2.1.3. Nucleotide Excision Repair

Nucleotide excision repair (NER) is an essential and highly efficient mechanism that eliminates a wide spectrum of lesions from DNA. NER recognizes various kinds of DNA damage such as adducts, DNA crosslinks, and UV irradiation lesions (cyclobutane pyrimidine dimers or 6,4 photoproducts). The NER system consists of more than 30 different proteins that participate in a coordinated and multi-step pathway to detect and remove the lesions [24]. There are two main NER mechanisms, the transcription-coupled NER (TC-NER), in which lesions are detected by RNA polymerase II during transcription, and the global-genome NER (GG-NER), where lesions are detected throughout the genome [25].

NER steps begin with recognizing the DNA damage by XPC-HR23B, or lesions such as cyclopyrimidine dimers, induced by UV light. Then, TFIIH is recruited to the lesion through specific protein–protein interactions. Two helicase subunits of TFIIH, XPB, and XPD are essential to unwinding the double helix. XPC does not directly interact with the lesion, but XPD directly interacts with the lesion through its translocation on the DNA. XPE (DNA damage-binding protein 2, DDB2) recognizes and binds to UV-induced damage, facilitating the XPC binding [26]. Then, it binds the scaffold XPA-RPA, and the XPG and XPF-ERCC1 nucleases are recruited. XPF-ERCC1 incises the DNA 5′ to the lesion, replication proteins initiate gap-filling synthesis, and after a 3′ incision by the XPG nuclease, the gap-filling is complete [27]. NER is critical for repairing bulky DNA lesions in DNA and defects of certain components can cause pathologies such as Xeroderma Pigmentosum, trichothiodystrophy, and Cockayne syndrome, among others [28,29]. Genetic alterations or mutations in NER genes are associated with cancer risk. Therefore, polymorphisms in NER genes have also been found in diverse types of malignancies, such as ovarian, breast, and lung cancer [30,31]. NER alterations are related to a response to cisplatin treatment [32]. NER is involved with different platinum-analog-induced DNA lesions, and associations between NER and platinum-compound responses have been reported in several tumors. The inhibition of this pathway may be a promising tool in cancer treatment [33,34].

#### 2.1.4. Mismatch Repair

The mismatch repair system (MMR) is the most relevant repair mechanism for maintaining genome stability. Deficiencies of the MMR are responsible for genomic instability (a cancer cell hallmark), and microsatellite instability (MSI). The variation in the lengths of the microsatellite repeats is known as MSI. MMR is a post-replicative DNA repair mechanism that corrects base–base mismatches and insertion–deletion loops [35]. MMR is integrated by a group of eight proteins in humans, homologs of the E. coli components MutS and MutL. The heterodimers MSH2-MSH6 detect single-base mismatches and dinucleotide insertion–deletion distortions, while MSH2-MSH3 recognizes 8-12 insertion–deletion loops. Then, the assembly with the MLH1-PMS2 complex leads to the repair of the DNA sequence [36]. MMR deficiencies are associated with Lynch syndrome or Hereditary Non-polyposis Colorectal Cancer, and the major gene responsible is hMLH1 which predisposes to colorectal, ovarian, and endometrial cancers [3]. The alterations of MMR lead to defects in DNA repair, increasing cancer risk. In addition, mutations in MMR genes can be related to drug resistance, such as cisplatin or temozolomide [37]. In addition, due to the importance of this DNA repair mechanism, different clinical trials on immunotherapy focused on MMR-deficient tumors have been developed [38].

#### 2.1.5. Double-Strand Break Repair

The molecule of DNA can be damaged by diverse deleterious agents such as ionizing radiation, chemotherapy, e.g., that can induce the most serious lesions called double-strand breaks (DSBs). DSBs can affect genome integrity. There are two pathways for DSB repair: homologous recombination (HR) and non-homologous end joining (NHEJ) [39]. HR occurs during DNA replication and the S and G2 phases of the cell cycle, thus it uses the complementary DNA strand as a template for the exact repair of the DSB, so it is considered error-free. However, NHEJ is available during all the cell-cycle phases and uses the microhomology of the two broken ends of the DNA to repair the DSB, so it is considered error-prone. Briefly, HR is a complex process that requires homologous sequences and starts with detecting the DSB by the MRN complex and CtIP (MRE11–RAD50–NBS1). RPA and RAD51 generate ssDNA tails and nucleofilaments. Then, 5′exonucleases assisted by BRCA1 degrade the DNA strand, followed by invasion facilitated by BRCA2 and synthesis using the sister chromatid as a template. The NHEJ process starts when the Ku70/80 heterodimer binds to the DSBs. DNA-PK phosphorylation occurs and Artemis processing, strand synthesis by Pol μ and λ, and ligation by ligase IV/XRCC4/XLF finally complete the mechanism. Other factors are APLF, PAXX, and XLF [40]. BRCA1 and BRCA2 are representative proteins, and their mutations are related to hereditary breast and ovarian cancer [41]. MRE11, RAD50, and NBS1 have been reported to have decreased in breast, ovarian, and colorectal cancer, and melanoma. RAD51 was increased in pancreatic cancer, leukemia, and sarcoma. DNA-PK and Ku70/80 were increased in breast, oesophageal, gastric cancers, and lung carcinoma [42]. Modifying DNA-repair proteins in cancers is essential for tumor cell survival. Specific anti-cancer treatment strategies including DNA-repair inhibitors are based on the overexpression of crucial components of the DSBs pathways in certain tumors such as RAD51. Several inhibitors for different steps of DSB repair have been developed and used in clinical trials, expanding therapeutic resources and truncating the ability of cancer cells to acquire resistance to treatment [43].

## 3. Wnt Signaling Pathway

The Wnt signaling pathways consist of both non-canonical and canonical routes. The non-canonical Wnt pathway operates independently of the β-catenin-T-cell factor/lymphoid enhancer-binding factor (TCF/LEF) axis and is divided into mechanisms known as the Wnt/Ca^2+^ pathway and the non-canonical Wnt-planar cell polarity pathway [44]. In contrast, in the canonical Wnt pathway, often called the Wnt/β-catenin pathway, β-catenin enters the nucleus, activating target genes through TCF/LEF transcription factors [8].

### The Canonical Wnt/β-Catenin Pathway

**Figure 1:**
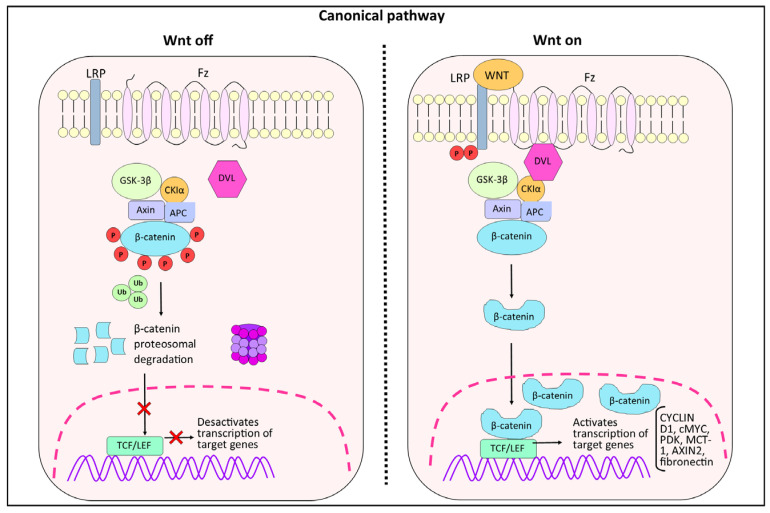
**Schematic representation of the canonical Wnt signaling pathway. Wnt Off:** in the absence of the Wnt ligand, β-catenin stability is controlled by the complex formed by GSK-3β, AXIN, CK1α, and APC (destruction complex). β-catenin is then phosphorylated and ubiquitinated for proteasomal degradation. **Wnt On:** In the presence of Wnt ligands, multimerization of Frizzled (Fz) receptors and LRP (lipoprotein receptor-related protein) co-receptors occur on the cell surface. The Dvl protein is recruited to the cell membrane to interact with Frizzled receptors, leading to interaction with the destruction complex. Wnt signaling is activated by binding to its receptor, which induces AXIN binding to phosphorylated LRP. β-catenin is then stabilized and binds to TCF in the nucleus to activate transcription of target genes. GSK-3β (glycogen synthase kinase-3β). AXIN (axis inhibitory protein). CK1 (casein kinase 1). APC (adenomatous polyposis coli). TCF (T-cell factor). LEF (lymphocyte-enhancing factor-1).

The main components of the canonical Wnt signaling pathway comprise Wnt family proteins, the receptors FZD (Frizzled) and LRP6, the Dishevelled protein (Dvl), β-catenin, T-cell factor (TCF), lymphoid enhancer factor (LEF), and the β-catenin destruction complex, which consists of adenomatous polyposis coli (APC), axin, glycogen synthase kinase-3β (GSK-3β), and casein kinase 1α (CK1α). Without a Wnt signal (Figure 1), β-catenin is bound by the destruction complex, leading to its ubiquitination and consequent degradation through the proteasome, mediated by β-TrCP. When a Wnt signal is present, Wnt ligands bind to the FZD and LRP5/6 receptors, activating the Dishevelled protein (Dvl) and inhibiting the destruction complex. This inhibition allows the cytoplasmatic accumulation of unphosphorylated β-catenin, which is then translocated into the nucleus, associated with TCF/LEF to promote the transcription of target Wnt genes [8,9].

## 4. Wnt/β-Catenin Crosstalk with DDR and Repair Pathways

### 4.1. Wnt/β-Catenin Role in DDR

**Figure 2:**
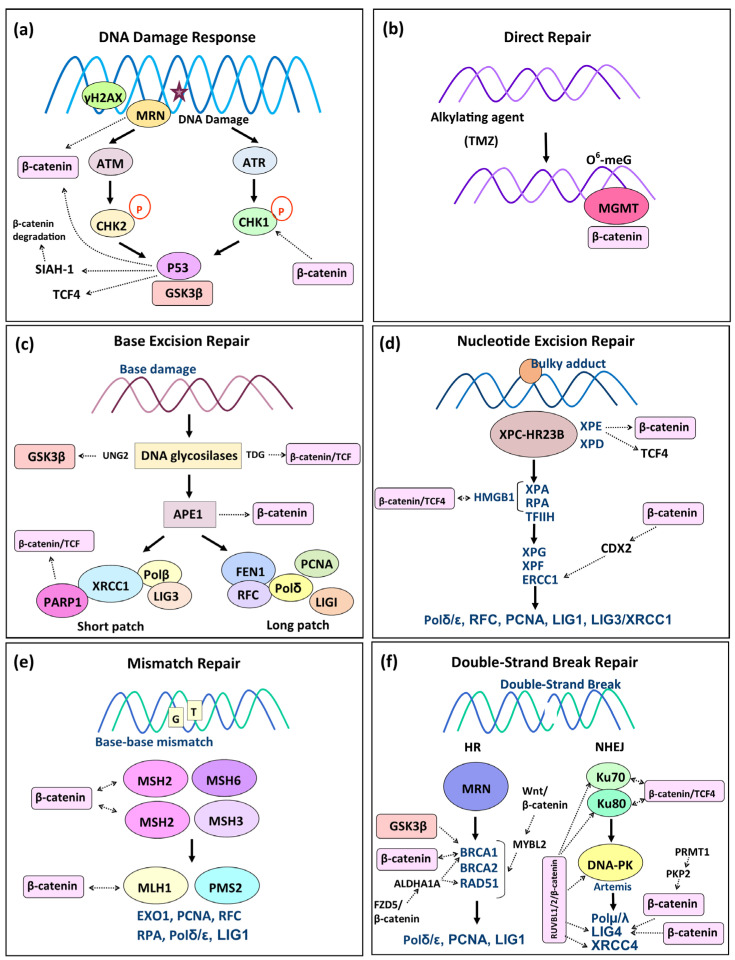
β-Catenin and DNA repair pathways. (**a**) DNA damage response (DDR). (**a**) DDR involves a signaling cascade from DNA damage sites, which ultimately activates the cell-cycle checkpoints to allow for repair or triggers cell death. ATM detects DNA double-strand breaks (DSBs), and ATR DNA single-strand breaks (SSBs). MRN complex is another important sensor of DNA damage. γH2AX amplifies the DDR signaling. The downstream effectors CHK1 and CHK2 phosphorylate p53, and p53 are important to activate the DNA repair or to initiate apoptosis. The specific participation of β-catenin and GSK3β in DDR is indicated in the figure. (**b**) Direct repair. This mechanism specializes in correcting the DNA damage induced by alkylating agents. The most frequent lesion is O6-methylguanine (O6-meG), recognized by O6-meG-DNA-methyltransferase (MGMT) and regulated by β-catenin. (**c**) Base excision repair (BER). BER is the most versatile DNA repair system for maintaining genome stability. The first step consists of the recognition step by specific DNA glycosylases. Two BER pathways are known: short patch-BER and long patch-BER. The DNA glycosylase excises the DNA damage, leaving an AP site (apurinic/apyrimidinic). AP endonuclease incises the AP site creating a nick, the DNA polymerase β replaces the damaged base in the short patch-BER. However, if the lesion consists of 2–11 nucleotides, PCNA and FEN1 endonuclease, which adds to the process known as long patch-BER. The specific participation of β-catenin in DDR is indicated in the figure, particularly with glycosylases. (**d**) Nucleotide excision repair (NER). NER is a multi-step system that recognizes a wide spectrum of lesions causing important distortions in DNA and involves the action of more than 30 different proteins. The global genome NER (GG-NER) identifies lesions throughout the genome. XPC-HR23B detects the lesion and additional factors such as TFIIH, XPB, and XPD are essential to unwinding the double helix. XPA-RPA, XPG, and XPF-ERCC1 nucleases are needed to initiate the incision 15-24 nucleotides away. Finally, the gap filling is completed by Polδ/ε, RFC, PCNA, LIG1, LIG3/XRCC1. The specific participation of β-catenin, TCF, and GSK3β in NER are indicated in the figure. (**e**) Mismatch repair (MMR). The heterodimer MSH2-MSH6 detects base–base mismatches and insertion–deletion loops (IDLs). MSH2-MSH3 recognizes larger IDLs. The heterodimer MLH1-PMS2 binds to the recognition complex and initiates the repair with the components EXO1, PCNA, RFC, RPA, Polδ/ε, and LIG1. The dotted lines indicate the participation of β-catenin. (**f**) Double-strand break repair (DSB repair). DSBs can be repaired by homologous recombination (HR) and non-homologous-end joining (NHEJ). HR acts after replication and begins when the MRN complex (MRE11-NBS1-RAD50) recognizes the DSB. BRCA1 binds to p53, BRCA2, RAD51, and the MRN complex. Resection of the 5′ DNA on either side of the DSB is BRCA1-dependent, resulting in the single-stranded DNA (ssDNA) exposure. BRCA2 localizes RAD51 to ssDNA regions to form a nucleoprotein filament capable of invading and binding homologous sequences. Then, DNA polymerases δ/ε and DNA ligase 1 end the repair of the lesion. The NHEJ system joins the two broken ends without homology. The heterodimer Ku70/Ku80 protects the DNA from exonuclease digestion and DNA-PK (DNA-dependent protein kinase). Artemis preserves the ends. DNA ligation is executed by the DNA ligase 4 (LIG4)-XRCC4 and end processing by DNA polymerase μ (Pol μ) and DNA polymerase λ (Pol λ). The participation of β-catenin, TCF4, and GSK3β is indicated with dotted lines.

The participation of Wnt signaling in DDR has been demonstrated (Figure 2a). Various studies have reported that the Wnt/β-catenin signaling pathway can influence DDR by regulating γH2AX, p16, p53, and p21 [45,46,47,48]. The Wnt signaling pathway is a P53 target, and β-catenin overexpression induces the accumulation of P53 in lung cancer cells [49]. Furthermore, high levels of wild-type P53 can downregulate β-catenin in human and mouse cells [50]. In addition, P53 can interact in the nucleus with GSK3β in neuroblastoma cells after camptothecin-induced DNA damage, suggesting that GSK3β has an independent function from the destruction complex [51]. P53 induces SIAH-1 (E3 ubiquitin ligase) for β-catenin degradation, thus reducing the activity of TCF/LEF transcription factors and contributing to cell-cycle arrest [52]. Besides regulating cellular responses to DNA damage, P53 can bind GSK3β, which can balance DNA repair and the cell cycle [53]. The Wnt/β-catenin transcription factor TCF4 is regulated by P53, and elevated P53 levels can downregulate TCF4 in colon cancer cells [54]. The Mre11–Rad50–Nbs1 (MRN) complex is another important component of the DDR in sensing DNA damage. Pasadi and colleagues reported that cisplatin exposure to human tumor cells significantly increased the expression of the MRN complex and stimulated Wnt/β-catenin signaling through increased β-catenin expression, activating the DDR and apoptosis. In addition, the downregulation of β-catenin in human cancer cell lines inhibited the phosphorylation of Chk1 induced by cisplatin treatment. These results show the importance of Wnt/β-catenin in maintaining genome stability [55]. CHK1 inhibition has been shown to increase the sensitivity of p53-deficient breast cancer cells to ionizing radiation, linking ATM to CHK1 [56]. These molecular interactions sustain the use of targeted therapies related to DDR and Wnt/β-catenin in cancer treatment [57,58]. Deregulation of DDR pathways increases genomic instability and carcinogenesis. Cancer cells’ intrinsic characteristics, such as DNA repair defects, turn them into vulnerable targets for specific inhibition [59]. Therefore, further investigation will be necessary to examine exhaustively the link between Wnt/β-catenin signaling and DDR.

### 4.2. Wnt/β-Catenin Role in Direct Repair

Agents with methylating properties, such as temozolomide (TMZ), have been extensively used in cancer chemotherapy. The main cytotoxic action of TMZ is based on the O6-meG induction to form the O6-meG/T base pair [60]. TMZ is used as a first-line therapeutic drug for the treatment of glioblastoma (GBM) [61]. The resistance to TMZ is associated with high expression levels of MGMT [62]. It has been reported that MGMT expression is inversely correlated to the survival of GBM patients treated with alkylating agents [63]. Extensive research has been focused on identifying molecules to inhibit MGMT [64]. However, MGMT inhibitors have shown promising preclinical results, although not significant benefits in the clinic for cancer patients treated with alkylating agents, causing researchers to explore MGMT inhibition in combination with cancer chemotherapy, or with the inhibition of other repair pathways [65].

It has been reported that activation of the canonical Wnt/β-catenin pathway induces MGMT expression, and inhibition of Wnt signaling augments the effects of alkylating drugs, restoring sensitivity in different cancers. In addition, immunofluorescence analysis on human colon cancer, glioma, medulloblastoma, and neuroblastoma cells showed co-localization of nuclear β-catenin and MGMT (Figure 2a) [66]. Moreover, Wnt/β-catenin signaling participates in invasiveness and chemotherapy (TMZ)/radiotherapy resistance in GBM [67].

### 4.3. Wnt/β-Catenin Role in Base Excision Repair

Uracil Nglycosylase (UNG) belongs to the uracil DNA glycosylase family and removes DNA-damage induced by 5-fluorouracil chemotherapeutic treatment [68]. Additionally, GSK-3β interacts and phosphorylates UNG2, Figure 2c [69]. Thymine DNA glycosylase (TDG) is highly expressed in colorectal cancer and interacts with the transcription factor TCF4 [70]. In addition, TDG promotes β-catenin/TCF transactivation [71].

APE1 functions as an AP endonuclease and its expression is altered in many types of cancer, including pancreatic, colon, lung, and ovarian, among other tumors [72]. In human pancreatic cells, reactive oxygen species (ROS) can modulate the Wnt/β-catenin pathway and APE1 inhibits Wnt/β-catenin by upregulating β-catenin when APE1 was downregulated by siRNA. In other terms, inhibiting APE1-suppressed cell growth, increased reactive oxygen species (ROS) levels, and upregulated β-catenin, thus demonstrating a crosstalk between APE1 and Wnt/β-catenin pathway in pancreatic cells [73]. Evidence suggests that Wnt/β-catenin can affect signaling target genes, such as cyclin D1 and c-myc [22].

PARP constitutes a DNA damage sensor activated in response to damaged DNA. Different reports showed the involvement of PARP-1 in BER [74,75]. PARP-1 is a co-activator of TCF-4/β-catenin and may participate in colorectal tumorigenesis [76]. Immunohistochemical analysis of colorectal tumors demonstrated that PARP-1 overexpression was associated with overexpression of β-catenin [77]. Evidence indicates that the activation of the Wnt/β-catenin signaling can induce resistance to PARP inhibitors (PARPi) [78]. The chronic inflammation of the colon epithelium can induce ROS and impair the Wnt/β-catenin and/or BER pathways [79,80]. The inflammation is associated with ROS production and oxidative DNA damage, which is repaired by BER pathway glycosylases such as MUTYH, including PARP activation [81].

Targeting BER by PARP1 inhibition in HR-deficient tumors has emerged as an important and efficient strategy [22]. Unfortunately, tumor cells can develop resistance to PARPi. The mechanisms implicated are diverse and combination with other therapies has been proposed to overcome such resistance [82]. For example, PARPi-resistant ovarian cells were sensitive to inhibition of Wnt signaling with pyrvinium pamoate (PP, approved by FDA), which downregulates β-catenin. PP with PARPi (Olaparib) decreased the tumor size in both ovarian cell lines and xenograft models. The study demonstrated that Wnt signaling mediates PARPi resistance in ovarian cancer and provides novel insights for using PARP and Wnt inhibitors [78]. Furthermore, the epigenetic gene regulation of FZD10 (Wnt receptor) contributes to Olaparib resistance in BRCA-deficient ovarian cancer cells by upregulating the Wnt/β-catenin pathway. This study also reports that inhibition of the Wnt/β-catenin pathway can overcome PARPi resistance [83]. BRCA-mutated ovarian cancers are frequently treated with PARPi and chemotherapy, but overcoming resistance is still a priority. The microRNA, miR-506-3p overexpression, can increase cisplatin and olaparib response in ovarian cancer cells, decreasing the level of β-catenin through the EZH2/β-catenin signaling pathway [84]. Despite advances in the mechanisms to overcome PARPi resistance, the Wnt/β-catenin pathway emerges as a potential target to explore and develop novel therapies in cancer.

### 4.4. Wnt/β-Catenin Role in Nucleotide Excision Repair

There is little evidence regarding the role of Wnt/β-catenin in the NER system (Figure 2d). DDB2 (XPE), is needed for the early steps of NER and is also a regulator of the Wnt/β-catenin signaling in colon cancer cells. DDB2 recruits β-catenin mediated by EZH2 (Enhancer of Zeste Homolog 2) a subunit of the Polycomb repressive complex 2 (PRC2), and promotes RNF43 activation, which restricts Wnt signaling. Therefore, a functional interaction between DDB2- and TCF4-binding chromatins in the RNF43 gene was also reported [85]. Caudal-type homeobox 2 (CDX2) has been widely recognized as a diagnostic biomarker for colorectal cancer (CRC), and CDX2 loss is also correlated with MMR deficiency [86]. A CDX2 role in DNA repair and Wnt signaling was demonstrated in human liver metastasis of CRC. CDX2 expression was associated with high expression of cytoplasmic β-catenin and nuclear APC. Elevated ERCC1 and CDX2 protein expressions were inversely associated with tumor size [87].

Chromatin remodeling is essential for repairing DNA damage. The high-mobility group box 1 protein (HMGB1) is a chromatin-associated protein involved in NER and other DNA repair mechanisms [88]. HMGB1 can bind to damage sites and cooperate with XPA-RPA proteins to facilitate chromatin modifications [89]. Interestingly, chromatin immunoprecipitation assays demonstrated the transactivation of HMGB1 mediated by a β-catenin/TCF4 complex, thus promoting DDR in esophageal squamous cell carcinoma (ESCC) consequent to exposure to ionizing radiation. Consequently, Wnt-induced radioresistance can be influenced by HMGB1. Therefore, Wnt inhibitors combined with radiotherapy could represent a promising treatment for ESCC [90]. There remains much to explore about the implications of Wnt/β-catenin in the NER system.

### 4.5. Wnt/β-Catenin Role in Mismatch Repair

MMR defects can increase β-catenin mutations and contribute to Wnt signaling activation, but mutations in β-catenin and increased DNA repair deficiencies can accelerate tumorigenesis (Figure 2e). Their interplay is important in certain tumor progression, such as colorectal and melanoma [91]. In colon epithelial cells from an MMR-deficient mouse model (MSH2-/-), excessive expression of active β-catenin by deregulation of Wnt signaling originated by loss of DKK1 (WNT inhibitor Dickkopf1), potentiate stem cell-like characteristics and abnormal proliferation [92]. Further studies are needed to establish the relationships of β-catenin with the MSH2 protein. Recently, a connection between β-catenin and MLH1 has been reported. Using transcriptome data from colorectal patients, the expression of MLH1 has been reported to significantly increase upon activation of the Wnt signaling pathway under various small-molecule drug-stimulating conditions. On the other hand, when the Wnt pathway was suppressed, MLH1 expression was downregulated. MLH1 and SET, a biomarker of MSI, were downregulated by the Wnt/TCF7 signaling axis in MSI patients [93]. Castiglia and colleagues reported that Wnt deregulation was associated with MMR deficiency in melanoma cell lines, and MLH1-deficient cells expressed nuclear β-catenin. In contrast, in MLH1-proficient cells the protein was completely absent [91].

In addition, the role of β-catenin location was highlighted in a group of patients with endometrial cancers by MMR status and PD-L1 (programmed death-ligand 1) expression. Lynch syndrome-associated cancers showed higher nuclear β-catenin expression than MLH1-hypermethylated and MMR intact tumors. PD-L1 expression was associated with β-catenin nuclear expression, which can also be considered a response biomarker in MMR-deficient patients [94]. Thus, the goal is to search for pan-tumor biomarkers to predict the responses to cancer therapeutic drugs and the translational role of β-catenin as a promising biomarker.

### 4.6. Wnt/β-Catenin Role in Double-Strand Break Repair

Wnt/β-catenin signaling can modulate DDR and is essential for repairing DSBs and preventing tumorigenesis (Figure 2f) [95]. The cisplatin treatment triggers Wnt/β-catenin by increasing β-catenin expression in cancer cell lines, and Wnt/β-catenin signaling and MRN complex crosstalk during the repair of cisplatin-induced DNA damage [55]. Li and colleagues reported the interaction between BRCA1 and β-catenin. BRCA1 interacts with β-catenin through the N-terminus containing the RING finger domain and the C-terminus with two BRCT repeats. The alteration of BRCA1 reduces the expression of nuclear β-catenin, which may participate in breast cancer pathogenesis [96]. Moreover, Wnt/β-catenin regulates MYBL2 and this pathway activates the expression of BRCA1, BRCA2, RAD51, and FANCD2. Resistance to DNA damage is often linked to the activation of the Wnt signaling. Otherwise, Wnt inhibition downregulates HR, promoting a BRCA-like state in Wnt-dependent cancer. This tumor subtype depends on activated Wnt signaling and is very sensitive to Wnt inhibitors, which is a new therapeutic strategy to overcome drug resistance. Moreover, Wnt and PARP inhibitors synergize to inhibit multiple Wnt-high cancers [97].

Glycogen synthase (GSK3) is a serine/threonine-protein kinase that regulates the Wnt/β-catenin pathway. Moreover, GSK3β also modulates HR DNA repair and the stemness of cancer cells, contributing to resistance against DNA-damaging chemotherapeutic agents and radiation [98]. A previous report had shown that the Wnt3a/GSK3β/Slug/Snail axis controlled the epithelial–mesenchymal transition (EMT) in association with Zeb1/2, vimentin, and fibronectin while coordinately repressing BRCA1 and BRCA2 expression in breast cancer cells exposed to DNA damage [99]. Nevertheless, GSK3β functions depending on Wnt/β-catenin signaling require additional investigation. Another study has demonstrated that GSK3β inhibition impaired HR efficacy, suppressed BRCA1 at mRNA and protein levels, and sensitized colorectal cancer cells to PARP and Top I inhibitors in replication-dependent DSB. A synergistic inhibitory effect of GSK3β depletion and PARP inhibition was detected in a panel of colon cancer cells [100].

A recent molecular profiling of homologous recombination deficiency (HRD) was performed in cholangiocarcinoma (CCA) cell lines and patient tumor samples. Mutations in homologous recombinational repair (HRR)-related genes were detected in CCA samples; however, their predictive value remains undetermined. Ten of the twelve cell lines showed changes in HRR-related genes, and five were HRD-positive, even though this result did not correlate with Olaparib sensitivity. Interestingly, functionally significant APC and β-catenin mutations were observed in CCA cell lines and tumor samples. These alterations were rare but exclusive to CCA with potential susceptibility to Wnt inhibitors [101].

Human Frizzled (FZD) molecules are 10 members (FZD1–FZD10) which contain a cysteine-rich domain (CRD). Extracellular Wnt binds to the CRD to initiate the canonical β-catenin pathway. Recently, FZD5 has been reported to induce stemness and HR repair in ovarian cancer cells resistant to DNA-damaging agents. In ovarian cancer cells, the knockdown of FZD5 downregulated the expression of active β-catenin through Akt. ALDH1A1, a downstream effector of the FZD5-β-catenin pathway, upregulates RAD51 and BRCA1, and increases HR repair. These findings indicate that targeting this pathway can sensitize ovarian cancer cells to therapy [102].

Regarding NHEJ, the expression of Ku70/80 has been reported to decrease after irradiation of head and neck cancer cells and β-catenin silencing. Furthermore, β-catenin silencing inhibited the irradiation-induced activation of Ku70/80 through the LKB1/AMP-activated protein kinase (LKB1/AMPK) signaling pathway [103]. Ku70 and Ku80 interact with the nuclear transcriptional complex TCF-4 and β-catenin. Ku70 coimmunoprecipitated with TCF-4 and physically interacts with the HMG box in HEK293 cells. The downregulation of Ku70 increased the association of β-catenin with TCF-4 and augmented the transcriptional activity, but the knockdown of Ku80 did not modify TCF-4 transcriptional activity. Therefore, Ku70 inhibits the β-catenin/TCF-4 transcriptional complex [104]. Eventually, DNA ligase LIG4 is upregulated by β-catenin in colorectal and intestinal cancer cells. LIG4 participates in Wnt signaling-induced radioresistance and is highly upregulated in human colorectal cancer cells, correlating with β-catenin hyperactivation. In addition, the Wnt pathway increases NHEJ activity in colon cancer cells, mediated by LIG4 transactivated by β-catenin [105].

Currently, research delves into radiotherapy resistance mechanisms mediated by NHEJ, HR, and Wnt pathways in cancer. Functional DNA repair assays have demonstrated that NHEJ and HR mechanisms increase DNA repair in high-grade serous ovarian carcinoma. A study has revealed that Wnt signaling activation is enough to promote PARP inhibitor resistance, increasing DNA repair capacity by NHEJ and HR, independent of BRCA2 reversion mutations [78].

The balance between HR and NHEJ can be regulated by the cell cycle and post-translational modifications (PTMs). One of the most frequent PTMs is arginine methylation, catalyzed by protein arginine methyltransferases (PRMTs). PRMT1 and PRMT5 are important regulators of DSB repair and are involved in balancing HR and NHEJ [106]. PRMT1 can promote methylation of plakophilin 2 (PKP2) to increase its interaction with β-catenin, leading to β-catenin stabilization and activating LIG4 in lung cancer cells. Thus, PKP2, β-catenin, and LIG4 are important players in NHEJ for radiation-induced DNA damage [107].

RUVBL1 and RUVBL2, also known as RUVBL1/2 are essential AAA+ ATPases that function as co-chaperones and are strongly associated with cancer. DTL-ubiquitination of RUVBL1 by Cul4-RING protein ligase (CRL4) induces the RUVBL1/2-β-catenin complex, which upregulates NHEJ gene expression through β-catenin-mediated transcriptional regulation. Thus, Wnt/ β-catenin signaling mediates radiation resistance in breast cancer cells, increasing the expression of the NHEJ components K70, K80, DNA-PKcs, 53BP1, LIG4, and XRCC4 [108].

## 5. Therapeutic Implications

The Wnt/β-catenin pathway is known to be activated in various tumors and has become a promising target for cancer treatment. The evidence has shown the inhibitory effects of Wnt/β-catenin in diverse cancer therapies, some of which are already undergoing clinical trials [109]. Antiparasitic drugs, most of them approved by the FDA, can suppress the Wnt/β-catenin pathway in many neoplasms, among them Niclosamide, Ivermectin, Mebendazole, Niclosamide, and Pyrvinium pamoate, which are under research to evaluate their effects in human tumors [110,111]. Quercetin, a flavonoid compound, has been shown to inhibit the Wnt pathway by interfering with the interaction between β-catenin and TCF-4 in colorectal cells, as well as to reverse multidrug resistance by targeting FZD7/β-catenin in hepatocellular carcinoma cells [112,113]. A wide variety of natural compounds that can inhibit the Wnt/β-catenin pathway have been described; however, the studies about most of these drugs remain at the in vitro level. Other direct and indirect Wnt/β-catenin signaling inhibitors have been developed such as small molecule inhibitors (SMI), peptide inhibitors, antibody drug conjugates, antisense oligonucleotides, PROTACs (proteolysis targeting chimeras), combination therapy agents involving SMIs, and monoclonal antibodies. These inhibitors target their components through multiple approaches [57,109].

However, reports on the therapeutic implications of Wnt/β-catenin pathway inhibitors and DNA repair mechanisms are limited and summarized in Table 1. There is still ongoing research work to explore in this field. The implications of MGMT (DR) modulation through the Wnt/β-catenin pathway for cancer treatment, particularly GBM, and different compounds have demonstrated anti-tumor activity reversing the resistance to TMZ [114,115,116]. In addition, the use of inhibitors of DSB repair molecules can benefit cancer patients from toxicity, resistance, and recurrence [97,100,101,102,107,108].

Wnt/β-catenin pathway targeting presents challenges, because of its complex network and consequences in normal cellular processes and stem cells [129,130]. To prevent undesirable effects on normal cells, novel approaches such as nanoparticles have been proposed [131,132]. Therefore, it is important to continue deepening the study of this pathway’s interactions with DNA repair mechanisms and the intended meaning of regulating them for the therapeutic approach of various tumors. In addition, the bioavailability of Wnt inhibitors and their tolerance constitute a reason for attention. The general objective is to attain a balanced therapeutic effect in confectionery to mitigate potential risks. This overview’s strengths are in collecting the reported implications of the Wnt/β-catenin pathway in DNA repair systems by delving into the molecular interplay between them and the advances in our understanding of the implicated mechanisms. The limitations encompass the broad spectrum of Wnt/β-catenin pathway inhibitors beyond the scope of this review. Despite these limitations, our overview aims to provide novel insights into the field of knowledge of Wnt/β-catenin’s role in DNA repair.

## 6. Conclusions

**Figure 3:**
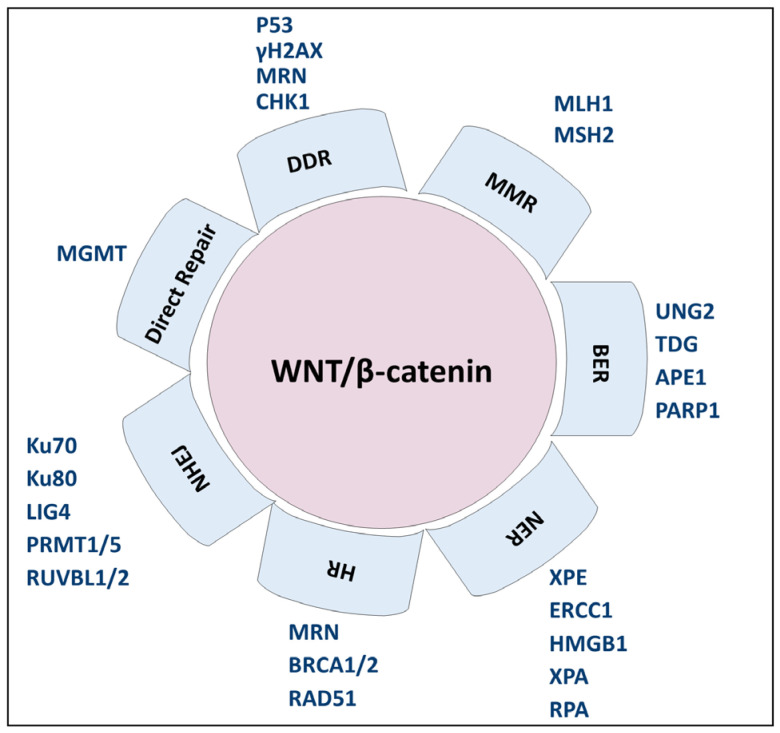
Scheme of the crosstalk between the Wnt/β-catenin pathway and DNA repair mechanisms. The main components of each DNA repair mechanism implicated in the Wnt/β-catenin pathway have been highlighted in the figure.

The Wnt/β-catenin pathway is a signaling cascade related to drug resistance, directly by activating target genes, or indirectly, e.g., by modulating DNA repair pathways. Both Wnt/β-catenin and DNA repair are involved in cancer hallmarks, which this makes them therapeutic targets. In recent years, great progress has been made in developing Wnt inhibitors. Still, despite the progress, no compound has yet been in commerce. Recognizing the challenges associated with the Wnt pathway is essential because of its involvement in normal cellular functions. Combining Wnt signaling inhibitors with DNA repair-related immunotherapies may sensitize cancers to radiation and other DNA-damaging agents. Therefore, the combination of drugs may reduce toxic effects and improve availability. Meanwhile, research is also directed toward the search for ways to increase therapeutic effects and develop personalized treatments in oncology. This review stresses the importance of applying innovative therapeutic strategies based on Wnt/β-catenin and DNA repair interplay (Figure 3). However, the implications of Wnt/β-catenin pathway in DNA repair from in vitro studies need further multidisciplinary research, despite representing a promising therapeutic approach against cancer. At last, the Wnt pathway inhibition alone or in combination with other therapies, such as DNA repair inhibitors or conventional therapies, can provide more tools for killing cancer cells and enhance therapies’ effectiveness.

## Figures and Tables

**Table 1:** **Wnt/β-catenin inhibitors** and **DNA repair: therapeutic implications**.

Compound	Wnt/β-Catenin Mechanism	DNA Repair Mechanism/Function	Cell Lines	Reference
Small molecule (compound 2)	Inhibits Wnt/β-catenin transcription.	(DDR) activates P53 transcription.	HCT-116, DLD-1, SW480, and 10.1-	Cheng, J. et al. [117].
Resveratrol	Wnt/β-catenin inhibition.	(DR) MGMT downregulation, TMZ-induced apoptosis.	U251, T98G, U138, A172, LN229 glioma cell lines.	Yang, H.C. et al. [118].
Cordyceptin	β-catenin downregulation.	(DR) MGMT inhibition and increases TMZ sensitivity.	LN18, T98G, LN229 and SHG-44 glioma cells.	Bi, Y. et al. [119]
Epigallocatechin gallate	It prevents β-catenin nuclear translocation and inhibits TCF1 and LEF1.	(DR) MGMT inhibition and increases TMZ cytotoxicity.	T98G cell line.	Xie, C.R. et al. [120]
Pyrvinium pamoate	Wnt/β-catenin inhibition and decreases β-catenin expression.	(DR) MGMT inhibition and increases TMZ sensitivity.	LN18, T98G, LN229, and U87MG GBM cells.	Li, H. et al. 2021 [121]
NBM-BMX (HDAC8 inhibitor)	Reduces β-catenin levels.	(DR) P53-mediated MGMT inhibition, increases TMZ sensitivity.	U87, U87R, A172, and A172R GBM cell lines.	Tsai, C.Y. et al. [122]
NCT503 (PHGDH selective inhibitor)	Wnt/β-catenin inhibition, by decreasing β-catenin levels.	(DR) reduces MGMT expression and increases TMZ sensitivity.	U87, U251, D54, A172, LN229, T98G, and U118 glioblastoma cell lines.	Jin, L. et al. 2022 [123]
E3330 and IWR-1 combination	IWR-1, Suppressed Wnt/β-catenin pathway.	(BER) E3330, APE1 inhibition, and cell growth inhibition.	SW-1990 and Panc-1 pancreatic cell lines.	Jiang, S. et al. [73]
PJ34 (PARP-1 inhibitor)	Inhibits β-catenin expression and TCF4 transcription.	(BER) Inhibits PARP1 and potentiates cytotoxicity.	HeLa and SiHa cervical cancer cells.	Mann, M. et al. [124]
AT-101 (derived from the cottonseed plant)	Inhibits Wnt/β-catenin signaling pathway by stimulating β-catenin degradation.	(BER) Redox inhibitor of APE1 and inhibits Bcl-2.	EC109 and CaES-17 esophageal carcinoma cell lines.	Que, F. et al. [125]
CBR-5884 (PHGDH inhibitor) and Olaparib	CBR-5884 inhibits ROS/Wnt/β-catenin pathway.	(BER) CBR-5884 acts synergically with Olaparib (PARP1 inhibitor), inhibiting cell proliferation, migration, and invasion. The mechanisms need further investigation.	A2780, OVCAR3, and ES-2 ovarian cancer cell lines.	Zhang, X. et al. [126]
1312 (Thiophene derivative)	Decreased β-catenin expression.	(BER) activates PARP and apoptotic cell death.	GES-1 (gastric cells), EC-9706, SGC-7901, and HT-29 (esophageal, gastric, and colon cancer cells, respectively).	Fu, L. et al. [127].
E7449 (small molecule)	Wnt/β-catenin pathway inhibition through TNKS1 and 2.	(BER, HR, NHEJ) PARP1/2 inhibitor, that potentiates the anti-tumor activity of TMZ and carboplatin. It also inhibits TNKS1 and 2.	Isogenic DT40 cell lines and MX-1 cells.	McGonigle, S. et al. [128]
ETC-159	Inhibits Wnt/β-catenin/MYBL2 pathway.	(HR) HR deficiency. ETC-159 with Olaparib combination increases DNA damage and senescence, sensitizing cancer cells to radiation.	HPAF-II, EGI-1, MCAS, CFPAC-1, PaTu8988T, or Panc 08.13 cells.	Kaur, A. et al. [97].
GSK3i (CHIR99021 HCl and LY2090314)	Wnt/β-catenin regulation.	(HR) GSK3i acts synergistically with PARP inhibitors (particularly simmiparib), impairs HR, and sensitizes colorectal cells.	HCC1937, HCT-15, RKO, HCT-116, HT-29, UWB1.289, SW480, SW620, UWB1.289 + BRCA1 cells.	Zhang, N. et al. [100].
C-7280948 (selective PRMT1 inhibitor)	Reduces β-catenin expression.	(NHEJ) reduces LIG4 expression, increases γH2AX and radiosensitivity.	A549 and H1299 cells.	Cheng, C. et al. [107].

## Data Availability

Not applicable.
